# Understanding neurocognitive recovery in older adults after total hip arthroplasty—neurocognitive assessment, blood biomarkers and patient experiences: a mixed-methods study

**DOI:** 10.1136/bmjopen-2024-093872

**Published:** 2025-01-28

**Authors:** Anahita Amirpour, Lina Bergman, Gabriela Markovic, Karin Liander, Ulrica Nilsson, Jeanette Eckerblad

**Affiliations:** 1 Neurobiology, Care Sciences and Society, Karolinska Institutet, Huddinge, Sweden; 2 Department of Clinical Sciences, Karolinska Institutet, Stockholm, Sweden; 3 Department of Rehabilitation Medicine, Danderyd University Hospital, Stockholm, Sweden; 4 Perioperative Medicine Intensive Care, Karolinska Universitetsjukhuset i Huddinge, Huddinge, Sweden

**Keywords:** NEUROLOGY, ORTHOPAEDIC & TRAUMA SURGERY, Old age psychiatry

## Abstract

**Objective:**

Delayed neurocognitive recovery, previously known as postoperative cognitive dysfunction, is a common complication affecting older adults after surgery. This study aims to address the knowledge gap in postoperative neurocognitive recovery by exploring the relationship between subjective experiences, performance-based measurements, and blood biomarkers.

**Design:**

Mixed-methods study with a convergent parallel (QUAL+quan) design.

**Setting and participants:**

The study reports results from 40 older adult patients (52.5% women; mean age 73, SD 6.7) scheduled for total hip arthroplasty at a hospital in Sweden.

**Outcome measures:**

Neurocognitive performance was assessed using a standardised test battery, neuroinflammation through blood biomarker analysis and postoperative neurocognitive recovery via semistructured interviews and the Swedish Quality of Recovery questionnaire.

**Results:**

Five patients were classified as having delayed neurocognitive recovery based on performance tests. Qualitative data revealed that most patients reported cognitive symptoms, particularly related to executive functions and fatigue. Psychological factors, including a sense of agency and low mood, significantly influenced cognitive recovery and daily functioning. Elevated inflammatory blood biomarkers were not detected pre- or postoperatively in patients with delayed neurocognitive recovery. The global postoperative recovery score was 40.9, indicating a low quality of recovery.

**Conclusion:**

Many patients reported subjective cognitive decline that was not corroborated by delayed neurocognitive recovery in the performance-based tests. Psychological factors were influential for neurocognitive recovery and should be routinely assessed. Future research should incorporate longitudinal follow-ups with performance-based measurements, fatigue assessment, evaluations of instrumental activities of daily living and subjective reporting, supported by a multidisciplinary team approach.

**Trial registration number:**

NCT05361460.

**STRENGTHS AND LIMITATIONS OF THIS STUDY**

- To our knowledge, this is the first mixed-methods study exploring performance-based measurements and subjective reports of postoperative neurocognitive recovery after orthopaedic surgery.
- We assessed neurocognitive performance with a test battery, explored postoperative neurocognitive recovery through semistructured interviews and measured the potential neuroinflammatory response with blood biomarkers.
- Results from 40 patients at a university hospital in Sweden are presented, a sample that may not be generalisable to other contexts.

## Introduction

 Delayed neurocognitive recovery (dNCR), formerly known as postoperative cognitive dysfunction (POCD),^1^ commonly affects older adults within a month post surgery^2 3^

Neuroinflammation and oxidative stress have been demonstrated to be a part of the mechanism of dNCR,^4 5^ and proinflammatory cytokines such as interleukin (IL)-6 and tumour necrosis factor α (TNF-α) enter the brain via normal or disrupted blood-brain barrier.^6^ Yet, at present there are no specific inflammatory biomarkers clinically validated for predicting or diagnosing dNCR.^5^ Moreover, fluctuations in tryptophan plasma levels have been suggested as a potential cause of postoperative fatigue, affecting serotonin 5-HT production and contributing to postoperative fatigue through 5-HT-synthesis resulting from changes in plasma amino acid levels.^7^

The recovery process from surgery is a multifaceted construct influenced by physical, psychological and social factors.^8^ Patients may regain their preoperative state or surpass it, reaching a high level of well-being and recovering lost functions.^8 9^ While perioperative research has emphasised overall recovery, the understanding of neurocognitive recovery in particular—what it entails, how it is experienced and its implications—remains ambiguous.

dNCR manifests with cognitive decline in memory, attention, processing speed and executive functions,^1^ and is linked to heightened disability risk.^10^ Traditionally, dNCR was assessed only through neurocognitive tests,^11^ but the updated nomenclature includes subjective cognitive decline (SCD) and daily function changes in the diagnosis.^1^ SCD, reported even without cognitive impairment, indicates elevated future cognitive impairment and dementia risk.^12^ However, perioperative research has primarily focused on quantitative measures of dNCR in the past decades, resulting in subjective reports being overlooked.

Therefore, this mixed-methods study aims to fill the current knowledge gap in postoperative neurocognitive recovery by integrating quantitative and qualitative data. By exploring performance-based measurements (neurocognitive test battery), blood samples (biomarkers) and how subjective reports on neurocognitive recovery (semistructured interviews and a patient-reported outcome) are experienced. We hypothesised that patients showing a decline in performance-based tests would have differing experiences in the interviews, and vice versa.

## Methods

### Study design and setting

This mixed-methods study had a convergent parallel (QUAL+quan) design^13 14^ and was conducted at a university hospital in Stockholm, Sweden. The mixed-methods design was qualitatively dominant, and we integrated both qualitative and quantitative approaches^15^ with the intention to provide an in-depth understanding of early postoperative neurocognitive recovery, following the Diagnostic and Statistical Manual of Mental disorders fifth edition criteria for mild/major neurocognitive disorders.^16^ We collected and analysed the quantitative and qualitative data separately, subsequently merged to identify any convergences, divergences or relationships between the two.

We obtained ethical permit (2019-02968) from the Swedish Ethical Review Authority on 19 June 2019, registered the study at ClinicalTrials.gov (NCT05361460), and published the study protocol.^17^ We recruited patients at the scheduled clinical preoperative visit, provided study information and obtained written informed consent from all patients, following the Declaration of Helsinki.^18^

### Study population

**Figure 1:**
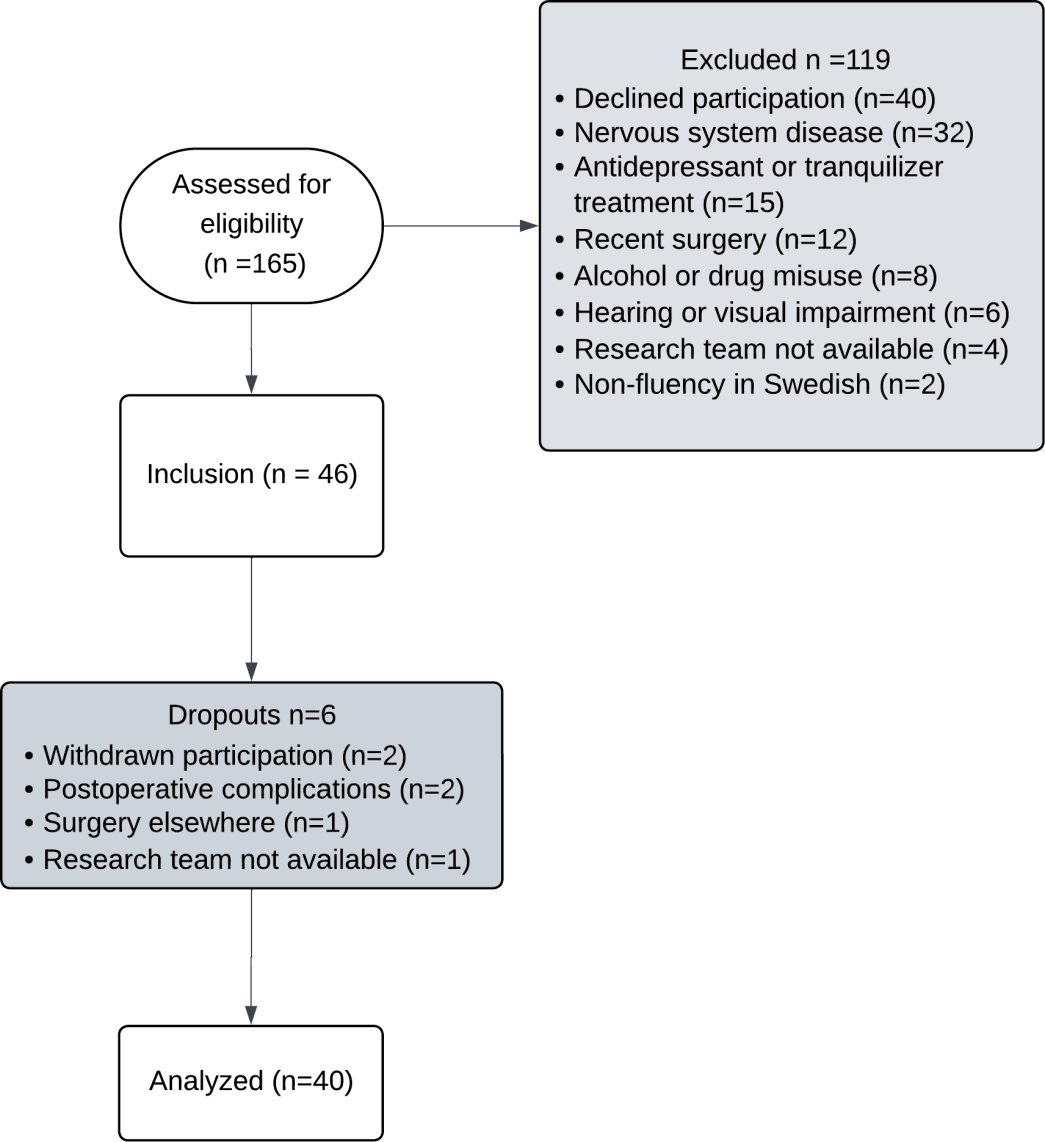
Flowchart of participants.

Between October 2019 and November 2021, we included 46 patients aged ≥60 years through convenience sampling. There were six dropouts (figure 1). Recruitment was extended by 18 months due to the COVID-19 outbreak. All potential eligible study participants were preliminary screened and approached by the fourth author. The patients were scheduled for total hip arthroplasty, and all patients underwent both the quantitative and qualitative data collection. Exclusion criteria were Mini Mental State Examination (MMSE)^19^ ≤ 22, nervous system disease, dependence on antidepressant or tranquilliser, alcohol or drug misuse, hearing or visual impairment, surgery in the previous 6 months and lack of fluency in Swedish.

### Outcome measures

We obtained demographic and perioperative data from patient records, including comorbidities, age, sex, MMSE score, pain intensity with numeric rating scale, education level, cohabitant status, American Society of Anesthesiologists classification, anaesthetic technique and duration, and duration of the surgery.

### Neurocognitive assessment

We measured neurocognitive performance with the International Study of Postoperative Cognitive Dysfunction (ISPOCD) test battery,^20^ administered by the fourth author, who was trained in neurocognitive testing. The battery includes four neurocognitive tests^20^:

1. **Visual Verbal Learning Test (VVLT**) measuring verbal episodic memory, based on Rey’s auditive recall of words, includes 15 words in three trials. A higher total word count indicates better scores.
2. **Concept ShiftingTask (CST)**, measuring visual mental flexibility, based on the Trail Making Test includes 16 circles in three trials. Less time and fewer errors indicate better scores.
3. **Letter-Digit Coding Test (LDC**), measuring executive attention, working memory and speed, based on the Symbol Digits Substitution Test during 60 s. High scores indicate better performance.
4. **Stroop Colour-Word Test (SCW**), measuring executive selective attention, includes 40 words in three trials. Less time and fewer errors indicate better scoring.

### Patient-reported outcome measurement

We assessed postoperative quality of recovery with the 24-item Swedish Quality of Recovery questionnaire (SwQoR-24). Each item measures various symptoms or discomfort related to surgery and anaesthesia such as pain, nausea, anxiety, sleep difficulties and fatigue. The patient rates these items on an 11-item scale, ranging from 0 (indicating none of the time) to 10 (indicating all the time). The range is from 0, indicating excellent quality of postoperative recovery, to 240, indicating poor quality of postoperative recovery. The patient is considered to have a good postoperative recovery if they have a global score less than 21 on postoperative day 14. The SwQoR-24 has been validated in a Swedish setting with postoperative patients.^21 22^

### Bloodborne biomarkers

We measured inflammatory biomarkers granulocyte-macrophage colony-stimulating factor (GM-CSF), interferon-ɣ (IFN-ɣ), IL-2, IL-4, IL-6, IL-8, IL-10 and TNF-α, and the non-inflammatory biomarker tryptophan at the preoperative visit, postoperative day 1 and on days 13–16 to assess its association with neurocognitive recovery. We took peripheral blood (11 mL whole blood) from the patient, centrifuged it and plasma was stored at −80°C until analysis. Tryptophan was measured using the standardised technique high-performance liquid chromatography. We analysed all blood samples in August 2023, with the Bio-Rad Bio-Plex Pro Human Cytokine 8-plex Assay #M50000007A.

### Procedure

The preoperative assessment at the orthopaedic clinic included a performance-based measurement using a standardised test battery (ISPOCD), blood sampling and SwQoR-24.The postoperative assessment on days 1–3 included blood sampling and SwQoR-24. On days 13–16, the postoperative assessment at the orthopaedic clinic included the test battery, blood sampling, SwQoR-24 and semistructured qualitative interviews. This timeline was selected to capture dNCR, which is manifested within 30 days after surgery.^23^

### Surgery and anaesthesia

The total hip arthroplasty surgery was carried out in accordance with normal clinical practice. Patients received spinal anaesthesia, either with 0.25 mL morphine (0.4 mg mL) and 2.8 mL bupivacaine (5 mg mL) at level L3–L4 or L2-L3, or with 3.5 mL bupivacaine (5 mg mL) only. Four patients underwent general anaesthesia with tracheal intubation using a combination of induction drugs such as alfentanil, propofol, fentanyl and a variation of neuromuscular-blocking drugs, and maintenance anaesthesia with sevoflurane.

### Qualitative data

Semistructured, face-to-face interviews were conducted 2 weeks after surgery. The interview questions covered cognitive functions, daily activities and overall mood, following an interview guide (online supplemental material 2). Each interview was audio-recorded and transcribed verbatim.

### Data analysis

#### Statistical analysis

Descriptive statistics are presented as means, SD, median score and completion times for the neurocognitive test battery. Wilcoxon signed-rank test was applied to assess changes in raw scores and completion times for the neurocognitive test battery. Normality of the data was assessed with Q-Q plots, histograms and Shapiro-Wilk test. A two-sided p-value of <0.05 was considered statistically significant. Cognitive performance changes were adjusted for practice effects and variability using age-matched nonsurgical controls; the z-scores were calculated to assess changes from preoperative to postoperative tests, with dNCR defined as a z-score of ≥1.0 on days 13–16 after surgery and z-score of <1.0 on days 13–16 indicated no decline according to the ISPOCD method.^20^ We followed the diagnostic rule for dNCR, meaning a decline in at least two subtests.^11^ We used IBM SPSS V.28 (IBM Corp, Armonk, New York, USA) for statistical analysis.

#### Qualitative analysis

Four authors (AA, GM, JE, LB) analysed the qualitative data. We applied Elo and Kyngäs’^24^ description of content analysis to the data, with a deductive and inductive approach. We initially chose a set of categories, that is, cognitive domains; attention and memory and executive functions based on theoretical framework,^25^ and our research objectives. These categories served as a structured matrix to code the data. As our analysis advanced, we recognised a recurring affective theme in the interviews. We openly coded these meaning units and categorised them as psychological factors, aligning with our research questions and acknowledging their influence on neurocognitive recovery. The analysis process involved several iterative steps. First, we read the verbatim transcribed interviews thoroughly. Then, we developed a structured categorisation matrix (online supplemental material 1) and reviewed and coded the data according to the categories and subcategories, and only extracted data that fit the final matrix.^24^ Finally, we held meetings regularly within our research group to achieve an agreement on data analysis.

#### Mixed-methods analysis

First, we analysed the qualitative and quantitative data sets separately. Then, we merged the results from the data sets by conducting a thorough side-by-side comparison, which is visualised in the joint display (table 1).^26^ The joint display comparison enabled us to assess for confirmation, discordance and expansion of the data sets, and draw meta-inferences.^26^ All findings were discussed within the research group. The initial proposed display was created by AA through an iterative process, with patterns, revisions and reviews conducted by LB and GM.

**Table 1:** Joint display presenting quantitative, qualitative and mixed-methods meta-inferences of domains

MM domains	Quantitative findingsNumber of patients with z-score ≥1.0, that is, delayed neurocognitive recovery on neurocognitive tests.	Qualitative findingsCodes and quotes	Meta-inferences
Executive functions	Stroop Colour-Word Test, n=12/40,Concept Shifting Task, n=10/40,Letter-Digit Coding Test, n=8/40.	*Performance awareness*So, you learn a lot of tricks, you stand in a corner, brace yourself against your back and stand on the leg you can put weight on. Yeah, then you can play around with the coffee maker. (P43)*Having a short fuse*So, I have a pretty short fuse, and I lose patience when things don't go smoothly … like when I can't put on my pants and stuff, so then I get angry. And then it might happen that a crutch ends up in the wall or something. (P31)*Not thorough as before and delaying action*I notice that’s not like me. I am very thorough about everything. But now, there are things everywhere, and, by the way, it’s hard to pick up. But I think, well, I'll do that later. But I haven't done it yet. (P08)*Motoric fatigue*I am tired, physically … if I go out and walk, as I have tried to do for the last three days … then I am quite tired afterwards …Yes, it’s time to lie down. And then I'm not really fit for fight … I don’t have much energy for the rest of the day. (P31)	In the performance-based results, only a small number of patients declined on the tests. Fatigue was not addressed in the neurocognitive tests; however, tryptophan levels were overall low in the total sample. Moreover, the qualitative data brought to light significant changes in patients' daily functioning, including changes in their performance at home or at work. Patients described new challenges in emotional regulation, where they would become frustrated or have anger outbursts on their family members. Some patients described a fatigue-like state, leading them to spend entire days in bed.
Attentionmemory	Visual Verbal Learning Test, n=4/40	*Doubting memory function*But, you know, it’s just that you start to think that you're not sure when you yourself stop noticing that you forget things. (P05)*Family member pointing out memory decline*If I have experienced some memory loss, it’s possible, it’s possible. Because our children said, “Dad, you won't remember this. It was like this.” (P01)*Feeling absent-minded*So right now, I can read and read and read, and still, I find myself stuck on the same sentence, and then and then it’s just as good to leave it (…)Uhm, concentration, I can't concentrate properly. (P10)	The test evaluated episodic memory at a specific point in time and demonstrated the lowest number of patients declining. The qualitative data showed that patients described attentional changes over time, with only a few acknowledging subjective memory decline or expressing family concerns about memory decline. Feelings of absent-mindedness and a lack of focus were identified as factors influencing both their memory and decision-making regarding the activities they chose to engage in or avoid.
Psychological factors		*Wanting to manage things independently*Sometimes it’s my dear wife … I become more easily irritated, perhaps. It has to do with her trying to be overly protective and fetch everything for me, and I think to myself, “I can handle this on my own,” and then I get slightly annoyed at trivial things that are not relevant. (P03)*Being in a bad mood and dependent on others*And the thing about being dependent on other people and … you don't want to bother people, even if they're your own sons, it feels like “God, how annoying I am.” And then I get in a bad mood. (P19)*Feeling low*I feel a bit depressed because I can't do anything, and not fix anything, not fetch anything, not pick up anything. (P08)*Brighter outlook*I think maybe I was grumpier before the surgery than after, because now it’s done. And now, well, theoretically at least, it can't get worse. Now it’s just going to get better. (P12)	While performance-based measures focus solely on the level of cognitive functions, they fall short in capturing the affective components. In the qualitative data, psychological factors were expanded on, with patients articulating the impact of factors such as the sense of agency, feelings of powerlessness stemming from dependence on others and adjustments to new physical limitations. These factors not only shaped their overall well-being but also significantly influenced their relationships and daily functioning. Conversely, a few patients shared a more optimistic perspective on life, attributing it mainly to the relief from previous pain.

#### Patient and public involvement

Patients and the public were not involved in the design, conduct, reporting or dissemination of this research.

## Results

This section starts with patient characteristics based on assessment data including biomarkers, followed by domain-level findings on executive functions, attention and memory, and psychological factors. In the ‘Discussion’ section, the integrated results are further expanded through a joint display (table 1).

### Patient characteristics and perioperative data

Six patients were excluded from the analyses because of withdrawn participation, postoperative complications, surgery elsewhere and the research team not being available, thus leaving 40 patients (figure 1). Patient characteristics and test results on a group level are presented in tables2  3.

**Table 2:** Patient characteristics

	Total sample (n=40)
Sex	
Men, n (%)	19 (47.5)
Women, n (%)	21 (52.5)
Age, years	
Mean (SD)	73 (6.7)
Min-max	60–87
Level of education	
Elementary school, n (%)	11 (27.5)
Upper secondary school, n (%)	13 (32.5)
Tertiary education, n (%)	16 (40)
Living situation	
Lives with spouse or adult children, n (%)	29 (72.5)
Lives with spouse and has home care, n (%)	1 (2.5)
Lives alone, n (%)	9 (22.5)
Lives alone and has home care, n (%)	1 (2.5)
Mini Mental State Examination	
Mean (SD)	28 (1.4)
American Association of Anesthesiologists’ physical status classification system	
I, n (%)	5 (13)
II, n (%)	18 (45)
III, n (%)	17 (42)
Comorbidities	
Heart disease (eg, hypertension), n (%)	24 (57)
Vascular disease, n (%)	9 (21)
Lung disease, n (%)	6 (14)
Kidney disease, n (%)	1 (2)
Diabetes, n (%)	5 (12)
History of cancer, n (%)	8 (19)
Autoimmune disease, n (%)	6 (14)
Type of anaesthesia	
Spinal, n (%)	36 (90)
General, n (%)	4 (10)
Duration of surgery, minutes (SD)	114.5 (32.4)
Duration of anaesthesia, minutes (SD)	188.5 (36.5)
Intraoperative bleeding, mL (SD)	348 (148.9)
Postoperative days at the hospital, mean (SD)	1.5 (0.6)
Preoperative pain, NRS, mean (SD)	5.4 (3.2)
Postoperative pain day 14, NRS, mean (SD)	2.1 (2.1)
Preoperative tryptophan, µmol/L, mean (SD)	43.8 (9.5)
Postoperative tryptophan, µmol/L, days 13–16, mean (SD)	41.9 (10)
Quality of recovery global score, days 13–16, mean (SD)	40.9 (28.4)
Postoperative opioid treatment, day 14	
Yes, n (%)	17 (43)
No, n (%)	23 (57)

NRSnumeric rating scale

**Table 3:** Summary of the patients’ raw scores and completion times on the neurocognitive tests

	Preoperative measurementMean (SD)Median	Postoperative measurementMean (SD)Median	P value*
VVLT total word count	22.3 (5.0)Med: 22.0	25.3 (5.9)Med: 25.5	<0.05
VVLT delayed recall, total word count	8.1 (2.6)Med: 8.0	9.1 (3.2)Med: 9.5	<0.05
CST, time (s), part C	38.8 (14.8)Med: 33.1	36.9 (13.5)Med: 35.4	0.49
CST, number of errors, part C	1.4 (2.7)Med: 0	1.1 (2.2)Med: 0	0.42
Letter-Digit Coding Test, score	27.4 (5.9)Med: 28	27.9 (7)Med: 30	0.39
SCW, time (s), part 3	51.4 (19.2)Med: 47.5	50.3 (22.1)Med: 43,8	0.15
SCW, number of errors	0.6 (1)Med: 0	0.9 (1,9)Med: 0	0.22

* Wilcoxon signed-rank test.

CSTConcept Shifting TaskSCWStroop Colour-Word TestVVLTVisual Verbal Learning Test

### Neurocognitive assessment

Among the 40 patients, 5 were classified as dNCR (z-score >1.0 in at least two subtests), with no statistical differences in anaesthetic factors or characteristics between those with/without dNCR. The mean scores and relevant completion times for each sub-test are presented in table 3.

### Patient-reported quality of recovery

On postoperative day 14, the patients’ postoperative recovery global score was mean 40.9 (table 2), indicating low quality of recovery. There were no differences in SwQoR-24 scores between those with/without dNCR.

### Bloodborne biomarkers

One patient did not have a preoperative inflammatory biomarker result, and three patients had missing results on the first postoperative day. The cytokines GM-CSF, IFN-γ, IL-10, IL-2 and IL-4 were undetectable in all patients, while IL-6, IL-8 and TNF-α were detectable but below 0 pg/mL. Tryptophan levels (table 2) were low both preoperatively and postoperatively in the total sample.

### Executive functions

Among the participants, n=12/40 declined on the SCW test, n=10/40 on the CST test and n=8/40 on the LDC test (table 1). Moreover, in the interviews (online supplemental material 1), the most significant and frequent problems the patients described were related to their executive functions. The main qualitative findings were associated to *problem-solving, emotional regulation, energisation and fatigue*.

These challenges manifested considerably when patients tried to resume their everyday activities at home or work, including meal preparation and initiating social contacts. While some patients dedicated effort to their physical rehabilitation, others refrained due to energy constraints, recounting days spent entirely in bed.

The patients described developing new strategies and skills to deal with the current changed form, where some patients learnt to carry the mug in another way, using a basket to carry the plate with food to the bed to eat, using a ladder with help from spouse, or if the patient was living alone, this also affected their strategy, doing the task independently:

And I have learned to walk, so that it works. But it was an effort I didn't think I would have to make. But it was the first time in these 50 years that I feel strained. (P01).

The effort to sustain energy to certain activities became particularly apparent in patients living with spouses or children as these family members assumed every task, from dressing to household chores. Patients struggling with these limitations often experienced emotional turmoil, expressing anger, impatience and frustration on realising their changed capacity for simple everyday tasks:

I've been a bit grumpy, I guess. I don't need to hide that. But no one has taken offense.

I've tried to be kind and nice, but sometimes you just snap a bit. (P10).

These issues with regulating emotions were previously unfamiliar to the patients and sometimes led to strained relationships, as some patients vented their emotions on their spouses.

The patients’ coping mechanisms varied, with some patients testing how far they could go in attempting presurgery activities such as leaving the house and go on a walk. Conversely, others embraced their current limitations, recognising the futility of certain tasks during this phase of recovery. The patients conveyed a profound sense of fatigue or lethargy, irrespective of what they did or following specific activities. This fatigue was articulated on either cognitive and motoric domain, or both:

The only thing I've managed is to go to the bathroom and take care of my needs and … yes, brush my teeth and things like that. […] I can handle such tasks, but nothing else. I don’t have the energy for it, I’m too tired. […] I couldn't even dress myself at first. My husband had to help me get dressed, you know. (P06).

In response to this fatigue, patients adopted alternative coping mechanisms. Some patients resorted to daytime sleeping while others avoided activities. This avoidance, distinct from their presurgical behaviour, was characterised by patients refraining from planned activities. For instance, they reported a shift from an intention to tidy up the house to do nothing at all. Similarly, they described avoiding interactions with friends or family members due to a lack of energy to engage in conversations.

### Attention and memory

In the VVLT, n=4/40 patients declined. The main qualitative findings were related to *subjective or family concerns of memory decline, sustained attention and mind wandering*.

Patients frequently described instances of forgetfulness, such as entering the kitchen or bathroom and subsequently forgetting their intended tasks. Some explicitly acknowledged memory decline, recognising pre-existing issues even before surgery. Patients who experienced forgetfulness occasionally questioned themselves, speculating whether such lapses existed before surgery.

Yes, I feel like I've had a really poor memory for a long time now. Because I've been anxious about the surgery, and that affects concentration a bit. And I haven't been feeling very well before either. (P07).

Others recognised their memory decline to ageing. For example, one patient expressed family concern, revealing that a family member had commented on his memory loss recently. As a result, the family member had taken over tasks the patient once handled independently. Consequently, the patient articulated he perceived a memory loss.

Some patients described how their minds wandered, especially during activities like reading or showering, leading to difficulties in sustaining their attention. As a result, they often abandoned the task. In contrast, others created adaptive strategies to manage their focus and memory, such as preplanning their medication routine and organising pills in specific containers.

### Psychological factors

The main qualitative findings were related to *sense of agency, powerlessness, physical limitations and future perspectives*.

In the interviews, some patients expressed a sense of relief and improved well-being post surgery, assigning it to the resolution of long-term pain that had accompanied every movement before surgery. This positive change had a notable impact on their mood as they reflected on their presurgery state characterised by persistent pain.

I feel much more positive now than right after the surgery, as I sense that the pain is heading in the right direction, and the mobility in the operated leg also feels much better, in that way. So, I feel that I am regaining a bit more zest for life compared to before the surgery. (P03).

On the contrary, other patients conveyed feelings of powerlessness and dependence on family members post surgery, particularly in managing daily activities. Despite their family members' well-intentioned efforts to protect them, this gave rise to annoyance. The patients had a desire to maintain a sense of agency even though their abilities had changed post surgery. This transition from independent functioning to reliance on others resulted in feelings of despair or a bad mood:

… To 110%. I don't want to be dependent … Yes, I become disheartened and a little angry, and … What should I say? … Just this being dependent, it’s … Yes, I want to do everything myself if I may say so. Control my day, or control and manage and so on. (P08)

Expectations for the future and the ability to function independently raised concerns, especially regarding the possibility of driving a car again. These worries about the future, coupled with doubts about improvement, led to mood disturbances such as irritability and feeling low.

I have a different way, a different temperament. I don't recognize myself. I am sometimes sad, and that’s not something I used to be. (P46)

Several patients spoke about the significant shift from being in control presurgery to a postoperative state where they felt a loss of control over their bodies, their capabilities and a sense of being different. This perceived loss of control within the healthcare system left patients feeling exposed.

## Discussion

We explored how 40 older adult patients experienced neurocognitive recovery after total hip arthroplasty and how this experience aligned or differed with neurocognitive assessment and biomarker results. Interestingly, no apparent differences were observed between those with detected dNCR and those without, whether in qualitative or quantitative data collection. Consequently, the data were presented at the group level.

In the neurocognitive tests, only five patients were classified as dNCR. However, more patients showed impairments in individual cognitive domains. This indicates that, although they did not meet the criteria for dNCR, they still experienced some degree of cognitive impact. Among patients classified as having dNCR, no specific subjective complaints or expressions of worry were reported during the interviews. Moreover, SCD was widely expressed by many patients in the interviews. The incongruence between the performance-based measurements and SCD is anticipated^27^ as the controlled neurocognitive test environment lacks external distractions compared with home or the workplace. Patients may demonstrate normal cognitive performance briefly during the test, but their day-to-day functioning could be compromised, leading to SCD,^28^ which became noticeable in the qualitative data. Furthermore, our findings align with previous research that discovered no correlation between cognitive performance and self-reported cognitive complaints.^228^,^30^ Nonetheless, subjectively reported data in perioperative research are often obtained with a variability of methods, such as questionnaires,^30^ phone interviews post surgery^29 31^ or a single-item binary question.^2^ The variability in data gathering poses a substantial challenge in consolidating findings and identifying comprehensive patterns. The diverse definitions and measurement approaches for SCD further complicate this task. In contrast, ageing research on SCD has primarily focused on symptom type and intensity, with a higher symptom burden increasing the risk of clinical progression.^32^

We found no association between inflammatory biomarkers and dNCR, consistent with other studies.^33^,^35^ As the inflammatory biomarkers were either undetectable or below 0 pg/L, they were excluded from data integration. Previous studies vary in their results when using inflammatory biomarkers to detect dNCR; these variations may be due to different types of surgery and different methods of analysing inflammatory markers. For example, a meta-analysis^33^ revealed an association between elevated C reactive protein levels in both postoperative delirium and cognitive decline. However, insufficient evidence was available to draw conclusions regarding IL-6, while IL-8, IL-10 and TNF-α showed no significant association with cognitive decline. Similarly, a recent systematic review^36^ noted elevated IL-6 levels within <12 hours postoperatively in older adults but found no such association for TNF-α. Another study focusing on older adults after hip fracture surgery indicated that glucocorticoid administration reduced levels of IL-6 and TNF-α.^37^ Perioperative administration of glucocorticoids, commonly used in orthopaedic surgery, and non-steroidal anti-inflammatory drugs, frequently prescribed for osteoarthritis,^38^ have been found to suppress cytokines, including IL-6 and TNF-α.^39^ Aligning with these findings, updated European guidelines on postoperative delirium advise against the use of biomarkers for the prediction or prevention of delirium in at-risk patients^30^. Nevertheless, it remains uncertain whether this recommendation extends to dNCR, posing implications of the design of future trials.

Tryptophan levels were consistently low in our patients, similar to the findings in a study of patients with cancer-related fatigue.^40^ Interestingly, mean tryptophan levels in other studies were higher: 74.4 µM in patients with cancer-related fatigue^41^ and 65 µmol/L in bariatric surgery patients.^42^ Besides the serotonin pathway, tryptophan is catabolized in the kynurenine pathway and plays a role in energy homeostasis. Changes in this pathway can be associated with low-grade inflammation,^41^ which is relevant to our patient group with osteoarthritis, a chronic inflammatory condition. Postoperative fatigue, characterised by persistent weakness or tiredness, is frequently overlooked and significantly impacts cognitive, behavioural and physical functions, often delaying the resumptions of daily activities after surgery.^43^ We found that two of the most frequently described symptoms in the qualitative data were lack of energy and lethargy impacting the patient’s daily functioning after surgery, aligning with previous research.^44^ However, we did not ask how their energy levels were before the surgery. Lethargy and lack of energy may be interpreted as fatigue which is not traditionally a component in neurocognitive tests even though it impacts cognitive functioning. Assessment of postoperative fatigue can be a helpful element, in addition to neurocognitive assessments, to predict postoperative recovery. Furthermore, each meaning unit from the qualitative data may not exclusively correspond to a singular cognitive domain but can in fact match to more than one, such as subjective complaints about attention could match with memory. Previous literature indicates attention, working memory and executive control share substantial similarities in their functional and structural neural correlates.^45^

Postoperative pain was well-controlled, as evidenced by the low pain scores. While some patients described an improved sense of well-being after experiencing pain relief following surgery, others expressed concerns about the future and the ability to function independently. The different coping strategies which the patient employed to resume daily activities align with earlier studies.^44 46 47^ In our study, patients described low mood, dependency on others and perceived loss of agency which impacted their daily functioning due to the surgery. Earlier studies^4447^,^49^ have found that psychological factors, specifically vulnerability factors like depressive and anxiety symptoms, affect postoperative recovery. These factors not only manifest behaviourally through avoidance behaviours but also have cognitive implications.^50^ Patients described how social support facilitated a smoother postoperative recovery process. Social support is related to improved global cognitive function, executive functions and memory.^51^ In our study, most patients resided with a spouse and received support in their daily activities, for example, help with dressing and cleaning. On the other hand, some patients expressed a sense of powerlessness due to their reliance on others. For them, depending on external support symbolised discomfort in seeking help. Therefore, clinical implications should include assessing surgical patients for emotional stress, such as depression and anxiety, as these factors are important predictors of postoperative recovery. Behavioural therapeutic interventions can be effective in addressing these concerns.^52^

The mean postoperative recovery score (SwQoR-24) was 40.9 on a group level, meaning they had a higher postoperative symptom burden and low quality of recovery, whereas a score <21 on postoperative 14, would indicate they had a good postoperative recovery.^22^ However, the quality of recovery score in the referenced study pertains to a day surgery unit with a mix of young and older patients, which may not be directly applicable to our group consisting of older adults with comorbidities.

To our knowledge, this is the first mixed-methods study exploring dNCR together with psychological factors after total hip arthroplasty. All participants underwent qualitative interviews, blood tests and neurocognitive tests, and our results present detailed descriptions of postoperative neurocognitive and emotional recovery. We acknowledge the limitations of this study. These include strict eligibility criteria which led to the exclusion of many patients and may have excluded frailer individuals, for example, those with nervous system diseases. Generalisability of our results is limited due to a small number of participants, and the convenience sampling is also a limitation. Further, this study lacked a standardised delirium assessment while patients were at the hospital and a preoperative depression screening. However, the SwQoR-24 does include items assessing anxiety and depressive symptoms. While we acknowledge the potential for bias with the same person conducting both tests and interviews, efforts were made to minimise bias by standardising the test procedure and instructions provided to all participants.

Future direction should involve multidisciplinary teams that bridge specialty, primary and social care services. Long-term follow-ups should include objective neurocognitive assessments, evaluations of fatigue and measurements of instrumental daily activities. Additionally, patients' subjective reports must be gathered in accordance with recommended terminology.^1^

## Conclusion

We found a disparity between subjective reports of neurocognitive recovery and performance-based measurements. Only five patients were classified as having dNCR; however, many patients described changes in their daily functioning due to cognitive and psychological symptoms. Our study highlights the complexity and breadth of postoperative neurocognitive recovery which extends beyond psychometric testing and blood samples.

## supplementary material

10.1136/bmjopen-2024-093872 online supplemental file 2

10.1136/bmjopen-2024-093872 online supplemental file 3

## Data Availability

Data are available upon reasonable request.
