# Supplementary material for: Understanding neurocognitive recovery in older adults after total hip arthroplasty—neurocognitive assessment, blood biomarkers and patient experiences: a mixed-methods study
Source: BMJ Open. 2025 Jan 28;15(1):e093872. doi: 10.1136/bmjopen-2024-093872 (PMC11781142; doi:10.1136/bmjopen-2024-093872)
Supplement: online supplemental file 2 [file bmjopen-15-1-s001.docx]

**Supplementary material**

1. **Procedure of content analysis**

| Categories | Subcategories | Codes | Quotes |
| --- | --- | --- | --- |
| **Executive Functions** | Problem-solving  Emotional regulation  Energization  Fatigue | Performance awareness  Feeling grumpy and snapping  Having a short fuse  Avoiding initiatives and others  Not thorough as before and delaying action  Mental fatigue  Motoric fatigue | And I have learned to walk, so that it works. But it was an effort I didn't think I would have to make. But it was the first time in these 50 years that I feel strained (P01)  So you learn a lot of tricks. You position yourself in a corner. Leverage against the back, and then you stand on the leg you're allowed to put weight on. Yes, and then you manage some things with the coffee maker (P43)  I've been a bit grumpy, I guess. I don't need to hide that. But no one has taken offense. I've tried to be kind and nice, but sometimes you just snap a bit (P10).  So, I have a pretty short fuse, and I lose patience when things don't go smoothly... like when I can't put on my pants and stuff, so then I get angry. And then it might happen that a crutch ends up in the wall or something (P31)  Yeah, I’ve mostly been lying in bed. That's what I've done. And I've been served food and everything, I haven't had to do anything. I have my grandchildren living with me, so he has helped a lot (P06).  I notice that's not like me. I am very thorough about everything. But now, there are things everywhere, and, by the way, it's hard to pick up. But I think, well, I'll do that later. But I haven't done it yet (P08).  But administrative tasks are no problem, but sitting down to write assignments, I don't have the concentration for that. I get too tired (P31).  The only thing I've managed is to go to the bathroom and take care of my needs and... yes, brush my teeth and things like that. // I can handle such tasks, but nothing else. I don’t have the energy for it, I’m too tired. // I couldn't even dress myself at first. My husband had to help me get dressed, you know (P06).  I am tired, physically ... if I go out and walk, as I have tried to do for the last three days…then I am quite tired afterwards…Yes, it's time to lie down. And then I'm not really fit for fight ... I'm don’t have much energy for the rest of the day. (P31) |
|  |  |  |  |
| **Attention and memory** | Subjective memory decline | Doubting memory function  Memory decline | But, you know, it's just that you start to think that you're not sure when you yourself stop noticing that you forget things (P05).  Yes, I feel like I've had a really poor memory for a long time now. Because I've been anxious about the surgery, and that affects concentration a bit. And I haven't been feeling very well before either (P07). |
|  |  |  |  |
|  | Family concern of memory decline | Family member pointing out memory decline | If I have experienced some memory loss, it's possible, it's possible. Because our children said, 'Dad, you won't remember this. It was like this' (P01). |
|  |  |  |  |
|  | Sustained attention | Losing the thread  Feeling absent-minded | Now it can be distracting around as well, I mean you can... What should I say? You lose the thread. If we have a study that we have read, a large section, so... //Now I haven't had the energy to participate and haven't had the energy to read up. I can't go through everything now, no. I don't do that (P06).  So right now, I can read and read and read, and still, I find myself stuck on the same sentence, and then and then it's just as good to leave it// Uhm, concentration, I can't concentrate properly (P10).  Today, I showered with the hearing aids on. It wasn't good (P01). |
|  |  |  |  |
|  |  | Lack of focus | These last few days, I have found it challenging. I have these magazines that I subscribe to, that I receive. So I haven't had the energy to read them properly. I haven't been able to focus on it (P39). |
| **Psychological factors** | Sense of agency  Powerlessness  Physical limitations  Future perspectives | Lack of agency  Wanting to manage things independently  Being dependant on others  Feeling low  Brighter outlook | But it's connected to the fact that I'm the kind of person who has a need for control, too, so that... And you don't have that when you surrender yourself to healthcare. (P05)  Sometimes it’s my dear wife... I become more easily irritated, perhaps. It has to do with her trying to be overly protective and fetch everything for me, and I think to myself, "I can handle this on my own," and then I get slightly annoyed at trivial things that are not relevant (P03)  …To 110%. I don't want to be dependent. Absolutely. //... Yes, I become disheartened and a little angry, and... What should I say? //... Just this being dependent, it's... Yes, I want to do everything myself if I may say so. Control my day, or control and manage and so on (P08)  And the thing about being dependent on other people and... you don't want to bother people, even if they're your own sons, it feels like 'God, how annoying I am.' And then I get in a bad mood (P19).  I feel a bit depressed because I can't do anything, and not fix anything, not fetch anything, not pick up anything (P08)  I have a different way, a different temperament. I don't recognize myself. I am sometimes sad, and that's not something I used to be (P46).  I feel much more positive now than right after the surgery, as I sense that the pain is heading in the right direction, and the mobility in the operated leg also feels much better, in that way. So, I feel that I am regaining a bit more zest for life compared to before the surgery (P03).  I think maybe I was grumpier before the surgery than after, because now it's done. And now, well, theoretically at least, it can't get worse. Now it's just going to get better (P12) |
|  |  |  |  |
