# Supplementary material for: Understanding neurocognitive recovery in older adults after total hip arthroplasty—neurocognitive assessment, blood biomarkers and patient experiences: a mixed-methods study
Source: BMJ Open. 2025 Jan 28;15(1):e093872. doi: 10.1136/bmjopen-2024-093872 (PMC11781142; doi:10.1136/bmjopen-2024-093872)
Supplement: online supplemental file 3 [file bmjopen-15-1-s002.docx]

**Supplementary material 2**

**Interview guide**

Open question to patients, encouraging them to speak freely about their experiences regarding memory, concentration, and recovery after hip replacement surgery.

- How do you perceive yourself from the time you were cared for in the recovery ward until now?
- How would you describe yourself since you underwent your hip replacement surgery?
- Do you recognize yourself after your hip replacement surgery? In what way? Please describe what you mean.
- How is your memory after the surgery compared to before? When and how did you notice any changes? Can you provide an example?
- Do you experience any changes in your concentration and attention? Can you elaborate on what you mean? Would you like to give an example or describe a situation?
- Have you experienced or do you experience changes in your mood after the surgery? Feel free to elaborate on your answer.
- Can you describe your sleep before and after the surgery? If it has changed, what do you think it might be due to? In what way is your sleep changed?
- Do you feel completely restored to your usual self, regarding your cognitive abilities after your surgery? If not, describe freely in what way and how. Can you describe the process?
- How do you perceive your ability to initiate your daily activities? Can you provide examples and describe it?
- How are your energy levels?
