# Supplementary material for: Understanding neurocognitive recovery in older adults after total hip arthroplasty—neurocognitive assessment, blood biomarkers and patient experiences: a mixed-methods study
Source: BMJ Open. 2025 Jan 28;15(1):e093872. doi: 10.1136/bmjopen-2024-093872 (PMC11781142; doi:10.1136/bmjopen-2024-093872)
Supplement: Supplementary file 3 [file bmjopen-2024-093872.reviewer_comments.pdf]

PEER REVIEW HISTORY

BMJ Open publishes all reviews undertaken for accepted manuscripts. Reviewers are asked to complete a checklist review form and are provided with free text boxes to elaborate on their assessment. These free text comments are reproduced below.

ARTICLE DETAILS

Title (Provisional)

Understanding neurocognitive recovery in older adults after total hip arthroplasty: neurocognitive assessment, blood biomarkers, and patient experiences - a mixed methods study.

Authors

Amirpour, Anahita; Bergman, Lina; Markovic, Gabriela; Liander, Karin; Nilsson, Ulrica; Eckerblad, Jeanette

---

VERSION 1 - REVIEW

**Reviewer**	**1**
**Name**	**Glumac, Sandro**
**Affiliation**	**Univ Hosp Split, Department of Anesthesiology and Intensive Care**
**Date**	**19-Sep-2024**
**COI**	**None**

This study entitled "Understanding neurocognitive recovery in older adults after total hip arthroplasty: neurocognitive assessment, blood biomarkers, and patient experiences - a mixed methods study" seems to have been generally well executed and written. Furthermore, I believe that this paper will be of great interest to the readers and very educational. However, I have a few remarks that require authors attention, and a few suggestions to further improve the quality of this important work.

Abstract

Please provide a short Background of your study (1 to sentences) before the Objective.

Keywords

Consider some additional MeSH keywords (e.g., Neuropsychological Tests) to readers easier identify your research.

Introduction

Please state the clear hypothesis of your study at the end of Introduction.

Methods

Surgery and anesthesia

Please add this subsection in the Methods. Here, describe in brief how the surgery and anesthesia were performed.

**Reviewer**	**2**
**Name**	**Zuo, Yunxia**
**Affiliation**	**Sichuan University, Anesthesiology**
**Date**	**11-Oct-2024**
**COI**	**None**

This manuscript presents a mixed-methods study on postoperative neurocognitive recovery in older adults following total hip arthroplasty. It combines quantitative assessments of cognitive function and inflammatory biomarkers with qualitative patient interviews to provide a comprehensive view of the recovery process. The findings reveal that while few patients showed delayed neurocognitive recovery based on tests, many reported cognitive decline and fatigue, suggesting a disconnect between objective measures and patient experience. The study also indicates that psychological factors play a significant role in recovery outcomes.

Major comments

1. The manuscript mentions a sample size of 40 patients but does not provide the rationale behind this number, which is essential from a statistical perspective to ensure adequate power.

2. The exclusion criteria are listed, but there is no detail on how these criteria were applied, which could potentially lead to selection bias.

3. The absence of a control group, such as patients undergoing other types of surgeries or a similar group of patients not undergoing surgery, limits the comparability and generalizability of the results.

4. The manuscript mentions data analysis using SPSS but does not specify the exact statistical tests used or how the level of significance was determined.

5. For the integrated analysis of mixed-methods data, a clearer description of how quantitative and qualitative results were combined is needed.

6. The manuscript refers to tables and figures that are not included in the version reviewed. Ensure that all mentioned tables and figures are present in the final manuscript.

7. The limitations of the study and how they might affect the interpretation and generalizability of the results need to be discussed.

Minor comments

1. There are instances where research is discussed without a corresponding reference number, such as when discussing the impact of surgery type and anesthesia choices on POCD.

2. The conclusion should be concise and directly address the research objectives and hypotheses.

3. The reference formatting needs to be uniformly checked to ensure compliance with the journal's citation standards.

VERSION 1 - AUTHOR RESPONSE

Reviewer #1 comments	Responses and comments
1. **This study entitled "Understanding neurocognitive recovery in older adults after total hip arthroplasty: neurocognitive assessment, blood biomarkers, and patient experiences - a mixed methods study" seems to have been generally well executed and written. Furthermore, I believe that this paper will be of great interest to the readers and very educational. However, I have a few remarks that require authors attention, and a few suggestions to further improve the quality of this important work.**	We thank you for the constructive and carefully considered feedback given to improve this manuscript.
2. **Abstract**<br>**Please provide a short Background of your study (1 to sentences) before the Objective.**	This has been added to the Abstract, page 2 row 25-29.<br>**Objective:** Delayed neurocognitive recovery, previously known as postoperative cognitive dysfunction, is a common complication affecting older adults after surgery. This study aims to address the knowledge gap in postoperative neurocognitive recovery by exploring the relationship between subjective experiences, performance-based measurements, and blood biomarkers.

3. **Keywords**<br>**Consider some additional MeSH keywords (e.g., Neuropsychological Tests) to readers easier identify your research.**	We have updated the keywords to MeSH keywords. Page 2, row 51-52<br><br>“**Keywords:** arthroplasty, replacement, hip, neurocognitive disorders, neuropsychological tests, postoperative cognitive complications
4. **Introduction**<br>**Please state the clear hypothesis of your study at the end of Introduction.**	This has been added, page 4, row 112-113.<br><br>“We hypothesized that patients showing a decline in performance-based tests would report differing experiences in the interviews, and vice versa. “
5. **Methods**<br>**Surgery and anesthesia**<br>**Please add this subsection in the Methods. Here, describe in brief how the surgery and anesthesia were performed.**	We appreciate the suggestion of adding surgery and anesthesia details. The total hip arthroplasty was carried out according to normal clinical practice. Since outcome related to surgical technique is not part of this study’s outcomes, we have not expanded this section further.<br><br>This has been added and revised, page 7, row 196-202<br><br>“Surgery and anesthesia<br>“The total hip arthroplasty surgery was carried out in accordance with normal clinical practice. Patients received spinal anesthesia, either with 0.25 ml morphine (0.4 mg ml) and 2.8 ml bupivacaine (5 mg ml) at level L3–L4 or L2-L3, or with 3.5 ml bupivacaine (5 mg ml) only. Four patients underwent general anesthesia with tracheal intubation, using a combination of induction drugs such as Alfentanil, Propofol, Fentanyl and a variation of neuromuscular blocking drugs, and maintenance anesthesia with Sevoflurane.”
**Reviewer #2 comments**	**Responses and comments**
1. **This manuscript presents a mixed-methods study on postoperative neurocognitive recovery in older adults following total hip arthroplasty. It combines quantitative assessments of cognitive function and inflammatory biomarkers with qualitative patient interviews to provide a comprehensive view of**	We thank you for the constructive and carefully considered feedback given to improve this manuscript.

**the recovery process. The findings reveal that while few patients showed delayed neurocognitive recovery based on tests, many reported cognitive decline and fatigue, suggesting a disconnect between objective measures and patient experience. The study also indicates that psychological factors play a significant role in recovery outcomes.**	
**2. The manuscript mentions a sample size of 40 patients but does not provide the rationale behind this number, which is essential from a statistical perspective to ensure adequate power.**	Since this is a mixed methods study, the main purpose is to gain new knowledge about the concept delayed neurocognitive recovery – rather than drawing statistical conclusions. This is also why the study is QUAL+quan, meaning, qualitatively dominant in its interpretations and findings.<br><br>The sample was made as a convenience sampling. We did not perform a formal sample size calculation. The sample size was determined at the time of when the study protocol was written (in 2019) and based on the earlier classification of postoperative cognitive dysfunction (POCD) and incidence of 17-25% (Silbert et al, Anesthesiology 2015).<br><br>We have updated the methods section, page 5, row 133-134.<br>"Between October 2019 and November 2021, we included 40 patients aged ≥60 years through convenience sampling, dropouts were 6 patients (Figure 1)."<br><br>And the discussions section, page 17, row 496-500<br>"We acknowledge the limitations of this study. Limitations include strict eligibility criteria which led to the exclusion of many patients, and may have excluded frailer individuals, e.g., those with nervous system diseases. Generalizability of our results is limited due to small number of participants and subjective reports. The convenience sampling is also a limitation."
**3. The exclusion criteria are listed, but there is no detail on how these**	We have added this detail to the manuscript page 5, row 135-136

	**criteria were applied, which could potentially lead to selection bias.**	"All potential eligible study participants were preliminary screened and approached by the fourth author."
**4.**	**The absence of a control group, such as patients undergoing other types of surgeries or a similar group of patients not undergoing surgery, limits the comparability and generalizability of the results**	We acknowledge the limitation of not having a surgical control group. However, the neurocognitive test results were matched with a nonsurgical control group<br><br>(See Data analysis – statistical analysis)<br><br>As this is a mixed methods study, the main purpose is not to draw generalizations but to enhance understanding and breadth of the phenomenon delayed neurocognitive recovery after surgery. This is why we applied the mixed methods design.
**5.**	**The manuscript mentions data analysis using SPSS but does not specify the exact statistical tests used or how the level of significance was determined**	We have specified the exact tests in manuscript, page 7, row 211-222<br>"Statistical analysis<br>Descriptive statistics are presented as means, standard deviations, median score, and completion times for the neurocognitive test battery. Wilcoxon signed rank test was applied to assess changes in raw scores and completions times for the neurocognitive test battery. Normality of the data was assessed with Q-Q plots, histograms and Shapiro Wilk Test. A two-sided p-value of <0.05 was considered as statistically significant. Cognitive performance changes were adjusted for practice effects and variability using age-matched nonsurgical controls, the z-scores were calculated to assess changes from preoperative to postoperative tests with dNCR defined as a z-score of ≥1.0 on day 13-16 after surgery, z-score of <1.0 on day 13-16 indicated no decline according to the ISPOCD method We followed the diagnostic rule for delayed neurocognitive recovery, meaning decline in at least two sub-tests We used IBM SPSS version 28 (IBM Corp., Armonk NY) for statistical analysis."
**6.**	**For the integrated analysis of mixed-methods data, a clearer description of how quantitative and qualitative results were combined is needed**	We have edited and added this information in the manuscript, page 8 row 239-246<br><br>"All findings were discussed within the research group. The initial proposed display

	was created by AA through an iterative process, with patterns, revisions and reviews conducted by LB and GM. "
**7. The manuscript refers to tables and figures that are not included in the version reviewed. Ensure that all mentioned tables and figures are present in the final manuscript**	We have double-checked the entire manuscript. In the manuscript, there is Figure 1 and Table 1-3 in this manuscript, each figure and table are referred to in-text. We also have supplementary material 1 and 2 and mentioned this in the manuscript as well.
**8. The limitations of the study and how they might affect the interpretation and generalizability of the results need to be discussed.**	We have updated the discussions section: Page 17 row 496-505<br><br>"We acknowledge the limitations of this study. This include strict eligibility criteria which led to the exclusion of many patients, and may have excluded frailer individuals, e.g., those with nervous system diseases. Generalizability of our results is limited due to convenience sampling and a small number of participants. Further,-this study lacked a standardized delirium assessment while patients were at the hospital and a preoperative depression screening. However, the SwQoRdoes include items assessing anxiety and depressive symptoms. While we acknowledge the potential for bias with the same person conducting both tests and interviews, efforts were made to minimize bias by standardizing the test procedure and instructions provided to all participants."
**9. There are instances where research is discussed without a corresponding reference number, such as when discussing the impact of surgery type and anesthesia choices on POCD**	We have double-checked the manuscript and updated the references where needed.
**10. The conclusion should be concise and directly address the research objectives and hypotheses.**	The conclusion has been revised:<br><br>Page 18 row 511-517<br><br>"We found a disparity between subjective reports of neurocognitive recovery and performance-based measurements. Only five patients were classified as having delayed neurocognitive recovery, however many

	patients described changes in their daily functioning due to cognitive and psychological symptoms. Our study highlights the complexity and breadth of postoperative neurocognitive recovery which extends beyond psychometric testing and blood samples. "
**11. The reference formatting needs to be uniformly checked to ensure compliance with the journal's citation standards.**	We have updated the references according to the journal's standards.
